# Characterization and Phylogenomic Implications of the Mitochondrial Genome of *Rhithrogena elasmaris* (Ephemeroptera: Heptageniidae)

**DOI:** 10.1002/ece3.73034

**Published:** 2026-02-12

**Authors:** Qi‐Yong Mu, Quan Zhou, Shook Ling Low, Yong‐Jing Zhao, Yong‐Xia Liu, Jun‐Yan Wu, Yong‐De Cui

**Affiliations:** ^1^ Guizhou Provincial Ecological Environmental Monitoring Centre Guiyang China; ^2^ State Key Laboratory of Lake and Watershed Science for Water Security, Institute of Hydrobiology, Chinese Academy of Sciences Wuhan China; ^3^ University of Chinese Academy of Sciences Beijing China; ^4^ Forest Research Institute Malaysia (FRIM) Kepong Selangor Malaysia

**Keywords:** codon usage bias, comparative genomics, mitochondrial genome, phylogenetic analyses, *Rhithrogena elasmaris*

## Abstract

This study reports the first complete mitochondrial genome of *Rhithrogena elasmaris* and provides a comprehensive analysis of its structural features and codon usage patterns. Combined with published mitochondrial genomes of 29 Heptageniidae species, we further evaluated genetic diversity, phylogenomic relationships, and differentiation patterns within the family. The mitochondrial genome of *R. elasmaris* is 15,326 bp in length with a GC content of 36.12%, and comprises the 37 typical mitochondrial genes. It shows a strong AT bias and clear codon usage preferences, with neutrality and PR2 plots indicating natural selection as the dominant evolutionary force. Simple sequence repeats are widely distributed, and tRNA structures are generally conserved despite frequent base mismatches. Comparative analyses demonstrate that gene order in Heptageniidae is highly conserved, although one copy of *trnM* has been lost in some species. Nucleotide diversity is relatively high (Pi = 0.223), with nad6 being the most variable protein‐coding gene and *cox1* the most conserved. Ka/Ks values < 1 across all 13 protein‐coding genes indicate strong purifying selection, with varying intensity reflecting functional constraints. Genetic structure and phylogenomic analyses support distinct subfamily level divergence within Heptageniidae. However, *R. elasmaris* and *Paegniodes cupulatus* exhibit mixed mitochondrial signals, suggesting possible incomplete lineage sorting or ancient mitochondrial introgression. The phylogeny supports the subfamily framework (Heptageniinae + [Ecdyonurinae + Rhithrogeninae]), with *Rhithrogena* forming a basal lineage within Rhithrogeninae. Divergence modeling indicates that Ecdyonurinae and Heptageniinae diverged first, followed by the split of Rhithrogeninae from Heptageniinae. This study enriches the molecular data resources for Heptageniidae and provides a refined framework for studying its systematics, evolutionary history, and ecological adaptation. Future work integrating nuclear genomic datasets will be necessary to further clarify speciation processes and adaptive evolution.

## Graphical abstract

This study presents the first complete mitochondrial genome of Rhithrogena elasmaris, revealing its structural features and codon usage patterns. Comparative analysis with 29 other Heptageniidae species showed high genetic diversity and strong purifying selection across protein‐coding genes. The phylogenomic results support a subfamilial framework of (Heptageniinae + [Ecdyonurinae + Rhithrogeninae]) and suggest possible mitochondrial introgression or incomplete lineage sorting in some lineages.

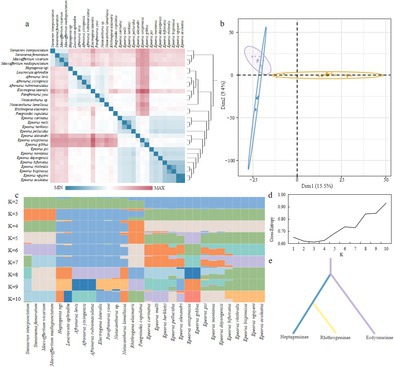

## 1 Introduction

*Rhithrogena elasmaris* (nomen nudum), belonging to the genus *Rhithrogena* within the subfamily Rhithrogeninae of the family Heptageniidae (Ephemeroptera), represents a newly discovered species from Hubei Province, China (Zhang 2021). To date, 10 species of this genus have been recorded across China, spanning both northern and southern regions (Zhang et al. 2021). Due to their ancient evolutionary history, limited dispersal capacity, high dependence on aquatic habitats, and relatively complete fossil records, members of Heptageniidae have become ideal materials for studying historical biogeography (especially the evolution of freshwater biota) and ecological adaptation in freshwater ecosystems (Vuataz et al. 2013). However, the scarcity of molecular resources, especially mitochondrial genome and whole genome information, has led to ongoing controversies regarding the phylogenomic relationships within Heptageniidae. This limitation hinders progress in reconstructing their evolutionary history, exploring mechanisms of ecological adaptation, and elucidating speciation processes. Therefore, generating high‐quality mitogenomic data is essential to clarify the phylogeny of Heptageniidae and to enhance our understanding of the diversification of East Asian freshwater insects.

The family Heptageniidae, one of the most diverse lineages within the order Ephemeroptera, exhibits considerable species diversity on a global scale and comprises 600 described species worldwide (Li, Lei, et al. 2021; Li, Zhang, et al. 2021). These species are widely distributed and play important ecological roles in freshwater systems. Historically, Heptageniidae classification was based predominantly on morphological traits. The first systematic framework was established by Eaton (1883–1888), followed by Needham and Betten (1901), who introduced the concept of subfamilies, including Heptageniinae. Subsequent revisions and phylogenomic analysis, such as those by Lestage (1917), who proposed Rhithrogeninae, further refined the classification. Jensen (1972) phylogenomically organized Heptageniidae into 4 subfamilies: Anepeorinae, Arthropleinae, Pseudironinae, and Heptageniinae. Subsequently, McCafferty and Edmunds (1979) established a new subfamily Spinadinae (Zhang 2021). These studies laid an important foundation for modern systematic research on the group.

However, morphology‐based phylogenomic inference presents inherent limitations. Subjective interpretation of diagnostic morphological traits and homoplasy has led to persistent controversies in subfamilial and generic classification. For example, the taxonomic status of the subfamily Heptageniinae has been repeatedly revised. Tshernova (1970) recognized 24 genera within this subfamily, whereas Webb et al. (2006) redefined its boundaries, transferring the tribe Compsoneuriini to the subfamily Ecdyonurinae. These revisions highlight the challenges of achieving phylogenomic stability through morphological data alone. The advent of molecular phylogenomics and genome sequencing technologies has provided powerful tools to resolve such taxonomic ambiguities. The view proposed by Wang and McCafferty (2004), based on molecular data, is widely recognized. Heptageniidae includes three subfamilies: Heptageniinae, Ecdyonurinae, and Rhithrogeninae.

Mitochondrial genomic data have since become a pivotal source of evidence for reconstructing the evolutionary history of Ephemeroptera (Zhang et al. 2008; Tang et al. 2014; Zhou et al. 2016; Gao et al. 2018; Ye et al. 2018; Xu et al. 2021; Li, Lei, et al. 2021; Li, Zhang, et al. 2021). Nevertheless, the internal phylogenomic structure of Heptageniidae remains unresolved, particularly at the subfamilial and generic levels. Disagreement persists regarding inter‐subfamilial relationships and generic boundaries. For instance, analyses based on the COI gene sequences (Webb et al. 2006) supported the topology (Rhithrogeninae + [Ecdyonurinae + Heptageniinae]), whereas a mitogenome‐based study of 13 protein‐coding genes (Xu et al. 2021) proposed an alternative topology: (Heptageniinae + [Ecdyonurinae + Rhithrogeninae]). Such discrepancies further underscore the complexity of refining the family's evolutionary framework. At the genus level, comparative mitogenomic studies have revealed structural variation and potential non‐monophyly in key lineages. For example, Ma et al. (2022) demonstrated instances of gene rearrangement and indicated that some subgenera may not be monophyletic (such as *Proepeorus*, *Belovius*, and *Iron*). Overall, the classification and phylogenomic relationships within Heptageniidae, particularly at the subfamilial and generic levels, require comprehensive molecular reassessment.

In this study, we sequenced, assembled, annotated, and characterized the complete mitochondrial genome of *R. elasmaris*, representing the first high‐quality complete mitogenome reported for the genus *Rhithrogena*. By integrating newly generated data with existing mitochondrial genomes of Heptageniidae, we performed analyses of genetic diversity, phylogenomic relationships, and species differentiation patterns. This approach not only offers a reliable perspective on the internal evolutionary dynamics of Heptageniidae but also provides robust molecular evidence for resolving long‐standing taxonomic controversies. The findings are expected to further refine the classification and phylogenomic framework of the family, thereby establishing a foundation for future research on the phylogeny, historical biogeography, and adaptive evolution of Heptageniidae.

## 2 Materials and Methods

### 2.1 Mitochondrial Genome Characteristics of *Rhithrogena elasmaris*

*Rhithrogena elasmaris* specimens were collected from Sancha River (104.5681° E, 26.7826° N), Guizhou, China, and preserved in 95% ethanol immediately after collection. No specific permits or permissions were required for this study, as the sample collection did not involve endangered or protected species and was conducted on publicly accessible land/public domain. Total genomic DNA was extracted from thorax tissue using the sodium dodecyl sulfate (SDS) method (Tian et al. 1999). DNA concentration and purity were measured using a NanoDrop One spectrophotometer (NanoDrop Technologies, Wilmington, DE), yielding a concentration of 84.31 ng/μL and an OD 260/280 ratio of 1.89. PCR amplification and library preparation were performed using Hieff NGS OnePot Pro DNA Library Prep Kit V4, followed by paired‐end (PE) 150 bp sequencing on the BGISEQ‐T7 platform.

Raw sequencing reads were filtered using fastp v0.20.0 (Chen et al. 2018), and the mitochondrial genome was assembled using GetOrganelle v1.7.7.0 (Jin et al. 2020). Genome annotation was carried out with MITOS2 (Bernt et al. 2013) using the RefSeq89 Metazoa reference database, and manual verification and adjustment were performed in Sequin v10.1 (Zhou et al. 2025). The mitochondrial genome was visualized using Chloroplot (Zheng et al. 2020), and tRNA secondary structures were visualized using tRNAscan‐SE 2.0 (Chan et al. 2021).

Thirteen protein‐coding genes (PCGs) were extracted using PhyloSuite v1.2.3 (Zhang et al. 2020). The effective number of codons (ENC) for these 13 PCGs, nucleotide composition at each codon position, AT‐skew, and GC‐skew were calculated using PhyloSuite v1.2.3 (Zhang et al. 2020). By referring to Zhou et al. (2025), relative synonymous codon usage (RSCU) was calculated in CodonW v1.4.2 (https://codonw.sourceforge.net/). Correspondence analysis (COA), Parity Rule 2 plot (PR2‐plot), ENC‐plot, and neutrality plot analyses were performed following the methodologies of Wang et al. (2025) and Zhou et al. (2025). All plots were generated by R 3.4.4 (Wang et al. 2025; R Core Team 2018).

Simple sequence repeats (SSRs) were identified using the MISA web tool (Sebastian et al. 2017), with the following parameters: mononucleotide repeats ≥ 8, dinucleotide repeats ≥ 5, and tri‐, tetra‐, penta‐, and hexanucleotide repeats ≥ 3. Following Zhou et al. (2025), compound microsatellites with a distance between SSRs of less than 100 bp were excluded from our analysis.

### 2.2 Comparative Analysis of Mitochondrial Genomes in the Family Heptageniidae

Additional mitochondrial genomes of 29 species were included, with one representative genome selected for each species in the family Heptageniidae (Table 1). These taxa comprise Heptageniinae (5 species), Ecdyonurinae (8), and Rhithrogeninae (16), and were downloaded from the NCBI GenBank database (Table 1). Specifically, Heptageniinae (5) includes *Heptagenia* (1), *Maccaffertium* (2), *Stenacron* (1), and *Stenonema* (1); Ecdyonurinae (8) includes *Afronurus* (3), *Electrogena* (1), *Leucrocuta* (1), *Notacanthurus* (2), *Parafronurus* (1); and Rhithrogeninae (16) includes *Paegniodes* (1), *Epeorus* (15).

**TABLE 1:** GenBank accession of Heptageniidae used in this study.

ID	Organism	Full length (bp)	Coding region length	Noncoding regions ratio
NC_084128.1	*Afronurus levis*	15,362	14,771	0.0385
MK642294.1	*Afronurus rubromaculatus*	15,519	14,768	0.0484
MK642297.1	*Afronurus yixingensis*	15,883	14,757	0.0709
NC_063607.1	*Electrogena lateralis*	15,378	14,722	0.0427
NC_065801.1	*Epeorus aculeatus*	15,451	14,756	0.0450
NC_065804.1	*Epeorus alexandri*	15,836	14,748	0.0687
MW381293.1	*Epeorus bifurcatus*	15,467	14,757	0.0459
NC_065802.1	*Epeorus bispinosus*	15,452	14,758	0.0449
NC_057491.1	*Epeorus carinatus*	15,338	14,770	0.0370
NC_057490.1	*Epeorus dayongensis*	15,609	14,756	0.0546
NC_065799.1	*Epeorus gibbus*	15,839	14,686	0.0728
NC_039612.1	*Epeorus herklotsi*	15,502	14,777	0.0468
NC_065657.1	*Epeorus melli*	15,490	14,754	0.0475
MW381295.1	*Epeorus montanus*	15,472	14,752	0.0465
MT679723.1	*Epeorus nguyeni*	15,466	14,773	0.0448
MW381296.1	*Epeorus pellucidus*	15,435	14,754	0.0441
NC_065805.1	*Epeorus psi*	15,654	14,751	0.0577
NC_065803.1	*Epeorus rhithralis*	15,447	14,757	0.0447
NC_065800.1	*Epeorus unispinosus*	15,849	14,695	0.0728
NC_065660.1	*Heptagenia ngi*	15,495	14,738	0.0489
MK642301.1	*Leucrocuta aphrodite*	15,428	14,760	0.0433
MK642303.1	*Maccaffertium mediopunctatum*	15,324	14,710	0.0401
MK642304.1	*Maccaffertium vicarium*	15,324	14,710	0.0401
NC_065661.1	*Notacanthurus lamellosus*	15,693	14,732	0.0612
MW381299.1	*Notacanthurus* sp.	15,524	14,747	0.0501
HM004123.1	*Paegniodes cupulatus*	15,715	14,712	0.0638
NC_011359.1	*Parafronurus youi*	15,481	14,769	0.0460
MK642305.1	*Stenacron interpunctatum*	15,330	14,711	0.0404
MK642306.1	*Stenonema femoratum*	15,332	14,715	0.0402

Whole‐genome alignments were conducted using MAFFT v7.037 (Tsukasa et al. 2018). Similarly, the 13 extracted PCGs using PhyloSuite v1.2.3 (Zhang et al. 2020) were aligned individually with MAFFT v7.037 (Tsukasa et al. 2018). The nucleotide diversity (Pi) values for the whole genomes (using the sliding window method) and for each gene were computed in DnaSP v6.0 (Julio et al. 2017) to evaluate single‐nucleotide polymorphism among species. Non‐synonymous (Ka) and synonymous (Ks) substitution rates were also calculated in DnaSP v6.0 (Julio et al. 2017) to assess selective pressures acting on the PCGs.

Gene‐order rearrangements among the Heptageniidae mitochondrial genomes were identified and visualized using PhyloSuite v1.2.3 (Zhang et al. 2020) and visualized using the ITOL web tool (https://itol.embl.de/) to detect potential inversions.

Sequence divergence across the Heptageniidae mitogenomes was analyzed using the annotated *R. elasmaris* mitochondrial genome as reference. GenBank files (.gb) were converted into the mVISTA format using a custom Python 3.10.1 script, and all 30 mitochondrial genomes were compared through the mVISTA server (https://genome.lbl.gov/vista/mvista/submit.shtml). Shuffle‐LAGAN global alignment algorithm, which accounts for inversions was selected, and genomic regions exhibiting more than 30% dissimilarity were interpreted as putative deletions (Zhou et al. 2025).

Collinearity and repetitive elements among the Heptageniidae mitochondrial genomes were further interrogated using MUMMER (Marçais et al. 2018; Zhou et al. 2025), using the *R. elasmaris* mitochondrial sequence as the reference.

### 2.3 Genetic Structure and Species Differentiation Analysis

Pairwise genetic distances were calculated in MEGA v7 (Sudhir et al. 2016) based on the p‐distance model with pairwise deletion.

Single nucleotide polymorphisms (SNPs) were identified using Stacks (Catchen et al. 2013) and subsequently filtered with VCFtools (Danecek et al. 2011). Species genetic structure was inferred using LEA (Frichot and François 2015), and principal component analysis (PCA) was performed with PLINK (Purcell et al. 2007).

Based on the filtered SNP dataset, DIYABC v2.1 (Cornuet et al. 2008) was employed to evaluate alternative divergence scenarios among the subfamilies Ecdyonurinae, Rhithrogeninae, and Heptageniinae. Informed by phylogenomic relationships and genetic clustering results, four competing divergence models were defined. A total of 1000,000 generations were run for each scenario in DIYABC to identify the most probable divergence history among the three lineages.

### 2.4 Phylogenomic and Evolutionary Analysis

Phylogenomic relationships among 32 species were reconstructed using two Ephemerellidae, *Cincticostella fusca* and *Torleya mikhaili* as outgroups. The optimal nucleotide‐substitution model was determined according to the Akaike Information Criterion (AIC) in ModelFinder (Kalyaanamoorthy et al. 2017). Bayesian inference was performed in MrBayes v3.2.7a (Ronquist et al. 2012) under the GTR + F + I + G4 substitution model. Two independent runs, each with four Markov chains, were executed for 1000,000 generations, sampling every 1000 generations. Convergence was assessed by examining the average standard deviation of split frequencies (< 0.01), and the first 20% of trees were discarded as burn‐in. The remaining trees were used to construct a majority‐rule consensus tree and estimate Bayesian posterior probabilities.

## 3 Results

### 3.1 Mitochondrial Genome Characteristics of *Rhithrogena elasmaris*

#### 3.1.1 Basic Features

**FIGURE 1:**
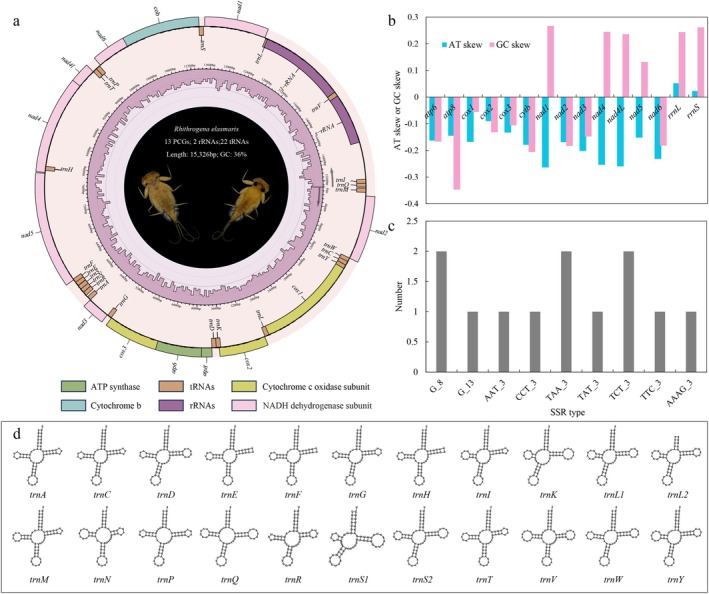
Characteristics of mitochondrial genome of *R. elasmaris*. (a) Circular depiction of the mitochondrial genome; (b) Nucleotide composition bias of PCGs; (c) Number of SSRs; (d) Secondary structures of tRNAs.

The complete mitochondrial genome of *R. elasmaris* is 15,326 bp in length (Figure 1a) with an overall GC content of 36.12%. It contains the canonical set of 37 mitochondrial genes, including 2 rRNAs, 22 tRNAs and 13 PCGs, with no free‐standing open reading frame (ORF) detected. The AT‐rich control region spans 571 bp and is located between *trnI* and *s‐rRNA*. The calculated AT‐skew and GC‐skew values range from −0.264 to 0.052 and −0.347 to −0.267, respectively (Figure 1b), indicating a strong compositional bias toward T and C nucleotides across most PCGs, consistent with intense natural selection.

A total of 12 SSRs were identified (Figure 1c) comprising 3 mononucleotide, 8 trinucleotide, and 1 tetranucleotide repeats. These SSRs were distributed across tRNA genes and non‐coding regions, with some large SSRs extending into adjacent protein‐coding regions.

The 22 tRNA genes range from 62 to 71 bp (total 1449 bp; Figure 1d). All tRNAs exhibit the typical cloverleaf secondary structure, and no loss of the dihydrouridine (DHU) arm or variable loop was observed. Base mismatches occur within the amino‐acid arm, TΨC arm, anticodon arm, and DHU arm.

Of the 37 mitochondrial genes, 26 are encoded on the L strand. A total of 19 intergenic overlaps were identified, ranging from 1 to 92 bp. The longest overlap is located between *trnH* and *nad4* (Table 2). Among the PCGs, nine terminate with the canonical TAA stop codon, whereas *cox1*, *nad3*, *cytb*, and *cox3* utilize the TAG stop codon.

**TABLE 2:** Mitochondrial genome of *R. elasmaris*. Arrangement and annotation.

Gene	Type	Start	Stop	Size	Continuity	Start	Stop	Strand
*trnM*	tRNA	17	82	66	2			L
*nad2*	PCG	140	1117	978	57	ATT	TAA	H
*trnW*	tRNA	1116	1183	68	−2			L
*trnC*	tRNA	1176	1237	62	−8			L
*trnY*	tRNA	1238	1304	67	0			L
*cox1*	PCG	1297	2841	1545	−8	ATC	TAA	H
*trnL2*	tRNA	2837	2902	66	−5			L
*cox2*	PCG	2908	3618	711	5	ATG	TAG	H
*trnK*	tRNA	3596	3664	69	−23			L
*trnD*	tRNA	3664	3730	67	−1			L
*atp8*	PCG	3731	3889	159	0	ATC	TAA	H
*atp6*	PCG	3886	4560	675	−4	ATA	TAA	H
*cox3*	PCG	4560	5348	789	−1	ATG	TAG	H
*trnG*	tRNA	5351	5415	65	2			L
*nad3*	PCG	5425	5769	345	9	ATA	TAG	H
*trnA*	tRNA	5768	5831	64	−2			L
*trnR*	tRNA	5862	5924	63	30			L
*trnN*	tRNA	5925	5986	62	0			L
*trnS1*	tRNA	5986	6051	66	−1			L
*trnE*	tRNA	6054	6117	64	2			L
*trnF*	tRNA	6116	6179	64	−2			L
*nad5*	PCG	6163	7869	1707	−17	ATT	TAA	L
*trnH*	tRNA	7915	7978	64	45			L
*nad4*	PCG	7887	9323	1437	−92	ATG	TAA	L
*nad4L*	PCG	9317	9613	297	−7	ATG	TAA	L
*trnT*	tRNA	9616	9679	64	2			H
*trnP*	tRNA	9680	9744	65	0			L
*nad6*	PCG	9762	10,265	504	17	ATT	TAA	H
*cytb*	PCG	10,265	11,401	1137	−1	ATG	TAG	H
*trnS2*	tRNA	11,400	11,469	70	−2			L
*nad1*	PCG	11,488	12,423	936	18	ATA	TAA	L
*trnL1*	tRNA	12,440	12,506	67	16			L
*rrnL*	rRNA	12,467	13,784	1318	−40			L
*trnV*	tRNA	13,786	13,856	71	1			L
*rrnS*	rRNA	13,856	14,637	782	−1			L
*trnI*	tRNA	15,209	15,273	65	571			L
*trnQ*	tRNA	15,271	14	70	−1			H

#### 3.1.2 Codon Usage Bias

**FIGURE 2:**
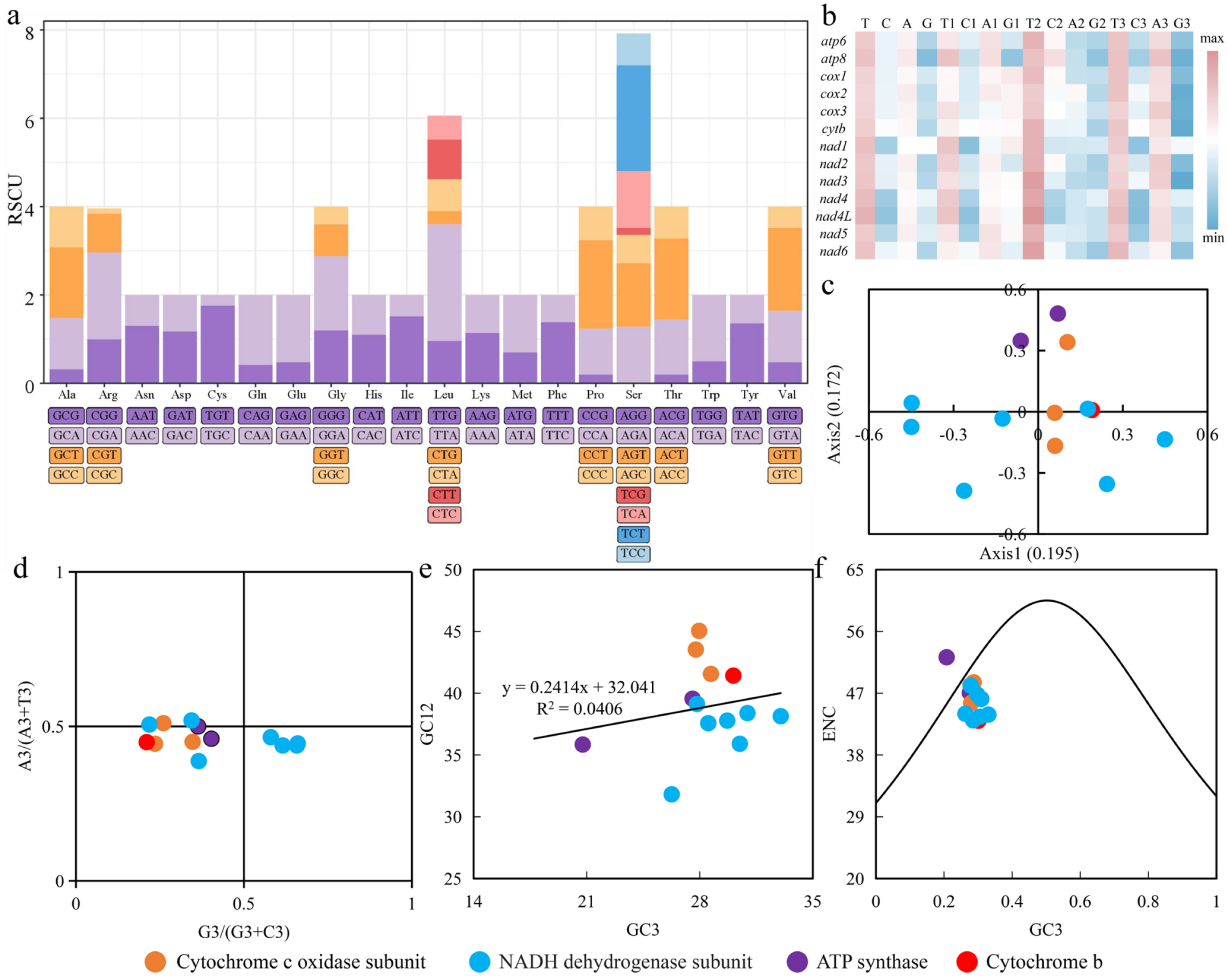
Codon usage bias analysis. (a) Relative synonymous codon usage within the PCGs; (b) Nucleotide composition metrics of each PCG; (c) Corresponding analysis based on RSCU; (d) Analysis of PR2‐plot; (e) Analysis of neutrality‐plot; (f) Analysis of ENC‐Plot.

A total of 27 high‐frequency codons with RSCU > 1 were identified in the *R. elasmaris* mitochondrial genome (Figure 2a; stop codons excluded). The highest RSCU (2.54) corresponded to UUA (Leu), whereas the lowest (0.00) was AGG (Ser). Among the RSCU > 1 codons, 2 end with G, 12 with U, and 13 with A, indicating a strong preference toward A/U‐ending codons. Across nearly all PCGs, the base composition followed the trend G < C, A < T, and AT > GC at both the whole‐gene and individual codon position levels, a pattern consistent with most published animal mitochondrial genomes (Figure 2b).

In the COA (Figure 2c), the first four principal axes, ranked by the proportion of variance explained, collectively accounted for 65.97% of the total variance in codon usage; axis 1 accounted for 19.5% and axis 2 for 17.2%. The wide scatter of genes along these axes indicates pronounced heterogeneity in codon‐usage patterns. Because the first two axes capture less than 50% of the total variance, codon‐usage bias cannot be ascribed to a single determinant; rather, it emerges from a complex interplay of mutation pressure, natural selection, gene length, and expression level, whose relative contributions vary across genes.

The PR2 plot analysis (Figure 2d) revealed that most PCGs deviate markedly from the central point (A_3_/(A_3_ + T_3_) = 0.5, G_3_/(G_3_ + C_3_) = 0.5). Most genes clustered in quadrant III, whereas none occupied quadrant I. Neutrality plotting analysis yielded a correlation coefficient of 0.2414 between GC_12_ and GC_3_ (Figure 2e), indicating that 24.14% of codon‐usage bias is attributable to mutational pressure, while 75.86% is shaped by natural selection. Additionally, most genes lie well below the standard ENC curve (Figure 2f). Collectively, these results indicate that natural selection exerts a predominant influence on the codon‐usage bias within the *R. elasmaris* mitochondrial genome.

### 3.2 Comparison of Mitochondrial Genomes of Heptageniidae

**FIGURE 3:**
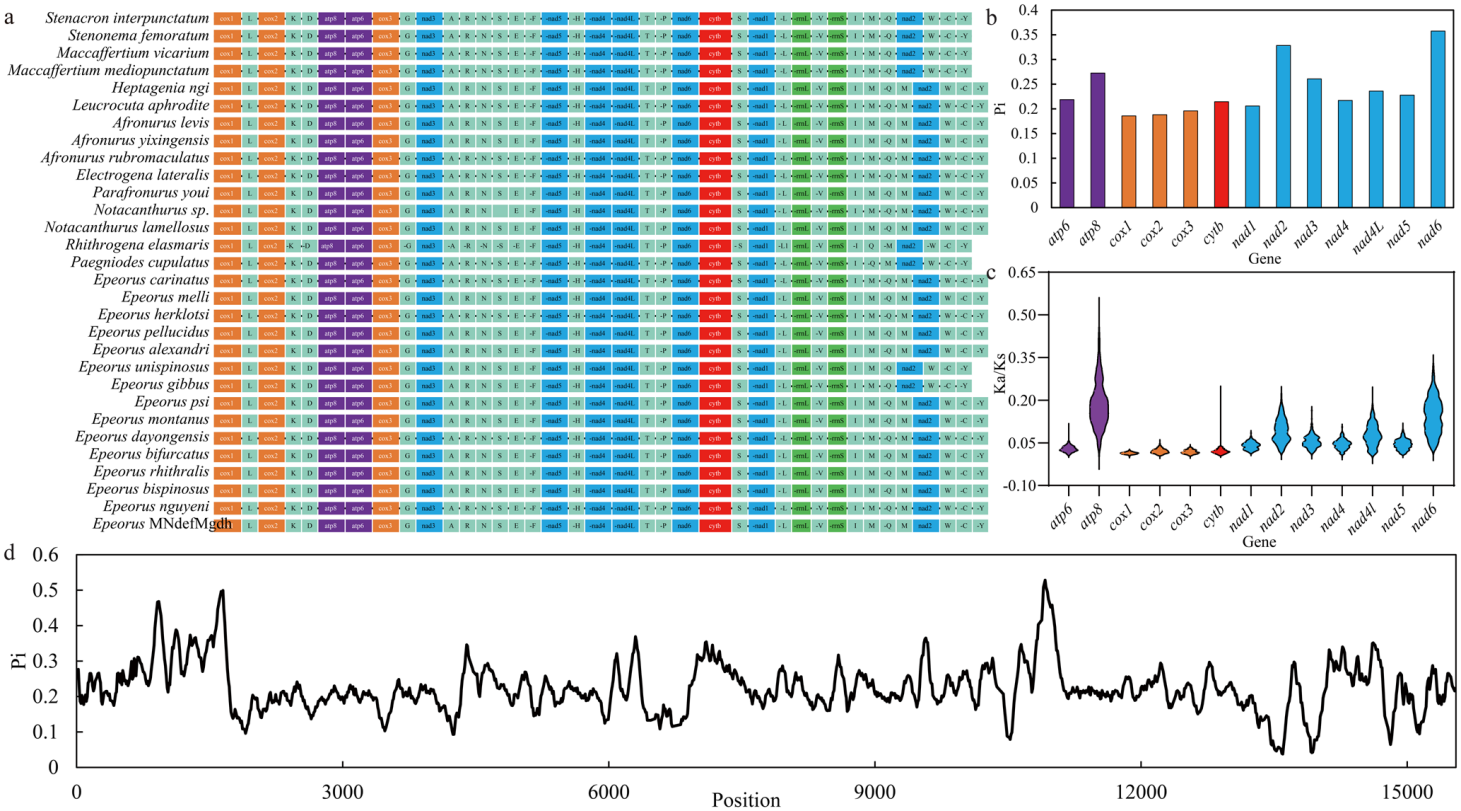
Comparison of mitochondrial genomic characteristics of Heptageniidae. (a) Gene order of Heptageniidae; (b) Nucleotide polymorphism in each PCGs; (c) The ratio of nonsynonymous to synonymous substitutions (Ka/Ks) of each PCGs; (d) Sliding window analysis of nucleotide polymorphism across the complete sequence.

The mitochondrial genomes of Heptageniidae species exhibit strong collinearity (Figure 3a), with no gene rearrangements detected. However, the multicopy *trnM* gene in the mitochondrial genome of Heptageniidae is currently undergoing elimination rather than insertion.

Overall, Heptageniidae mitogenomes exhibit high genetic diversity, with a genome‐wide average Pi of 0.223. Additionally, nucleotide polymorphism varies markedly across genomic regions. Specifically, untranslated regions (UTR, Pi = 0.182) were more conserved compared with PCGs (Pi = 0.238).

To further assess variation among individual genes, the Pi of genes was calculated separately for each of the 13 PCGs (Figure 3b). The overall average diversity among PCGs was Pi = 0.239, with *nad6* exhibiting the highest diversity (Pi = 0.357) and *cox1* showing the lowest (Pi = 0.186). This pattern suggests heterogeneous selective pressures acting on different mitochondrial genes. Within functional categories, the *nad* genes displayed the greatest variation (Pi = 0.262), implying relatively weaker environmental constraints during species divergence. In contrast, cox genes were the most conserved (Pi = 0.190), indicating stronger functional constraints and high evolutionary conservation.

The overall average Ka/Ks ratio for the 13 PCGs was 0.0620 (Figure 3c), substantially below 1, indicating that the mitochondrial genes of Heptageniidae are collectively under strong purifying selection to maintain the stability of their core biological functions. However, the intensity of selective pressure varied significantly among genes. The encoded products of *cox* genes are relatively more conserved, whereas those in the *nad* and *atp* categories exhibit greater variability. Specifically, the *cox1* gene had the lowest Ka/Ks value (0.0135), identifying it as the most conserved, while the *atp8* gene exhibited the highest Ka/Ks ratio (0.187), suggesting a faster evolutionary rate. Within the overarching framework of strong purifying selection, the mitochondrial PCGs in Heptageniidae demonstrate a diversified evolutionary pattern consistent with their functional importance.

**FIGURE 4:**
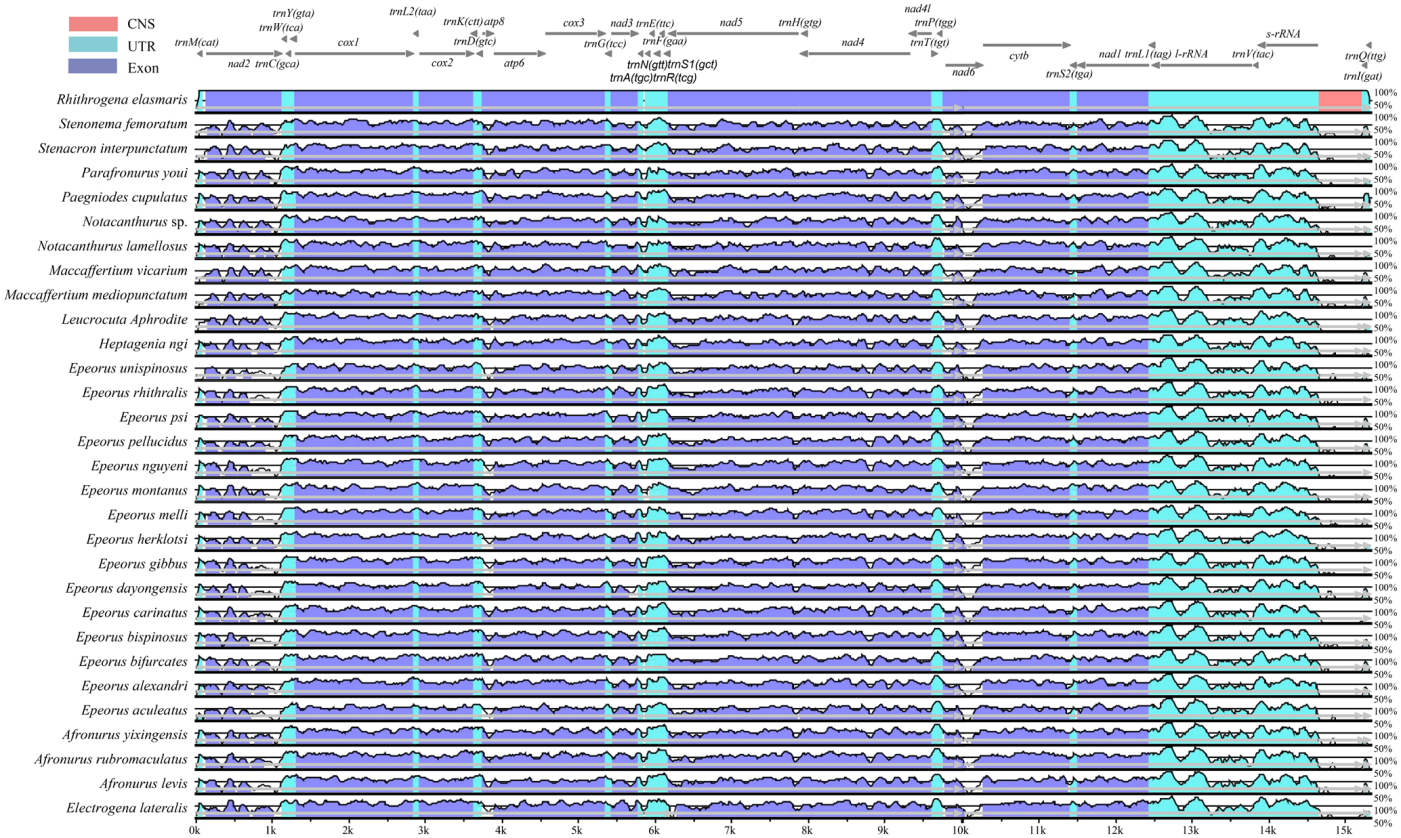
Sequence alignment of Heptageniidae.

Significant divergence was detected among the mitochondrial genomes of 30 Heptageniidae species (Figure 4), indicating clear phylogenomic differentiation within the family. The conserved non‐coding regions (CNS) exhibited the highest sequence variability, followed by exonic regions, whereas UTRs were the most conserved. Notably, the control region between *R. elasmaris* and other Heptageniidae showed striking sequence divergence, with sequence similarity below 30%.

**FIGURE 5:**
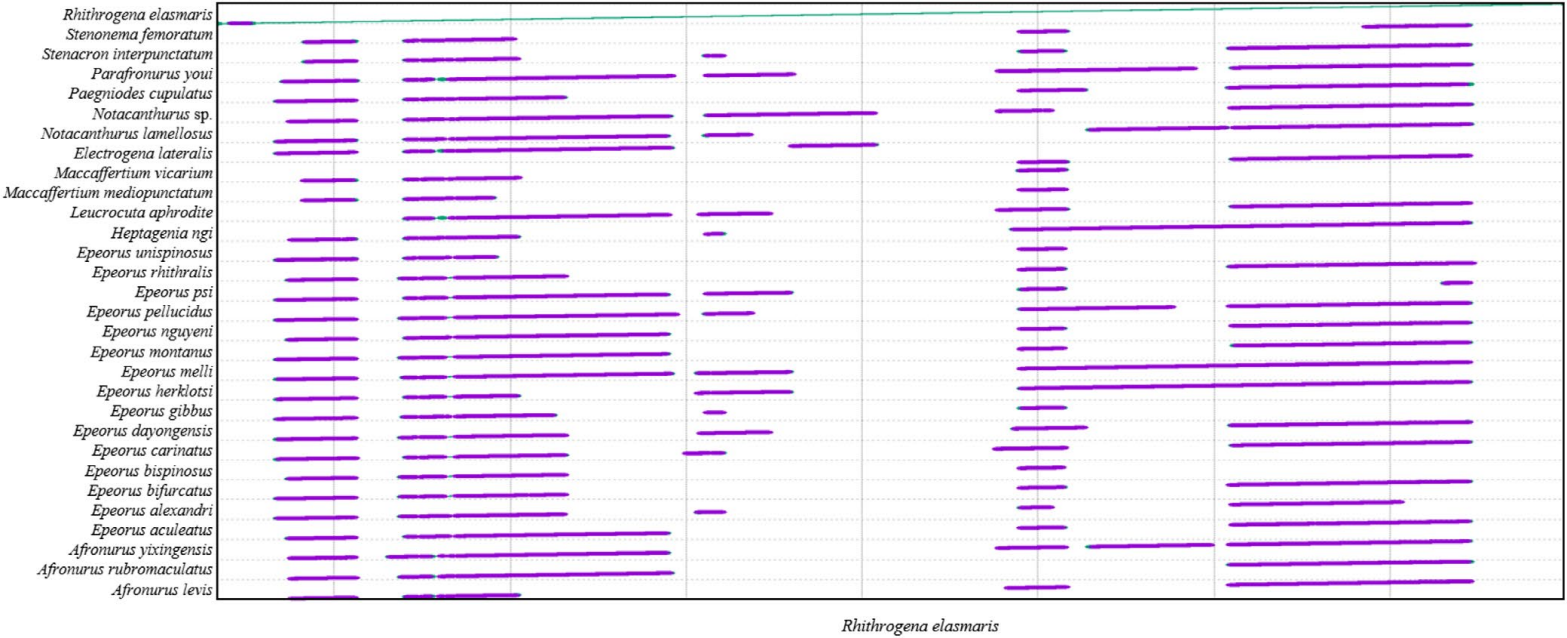
Sequence collinearity of Heptageniidae.

Despite these differences, the overall mitogenomic architecture of *R. elasmaris* remains highly conserved. As shown in Figure 5, regions of high sequence similarity retain clear homology and collinearity, and no evidence of large‐scale inversions or gene rearrangements was observed across the family.

### 3.3 Genetic Structure

**FIGURE 6:**
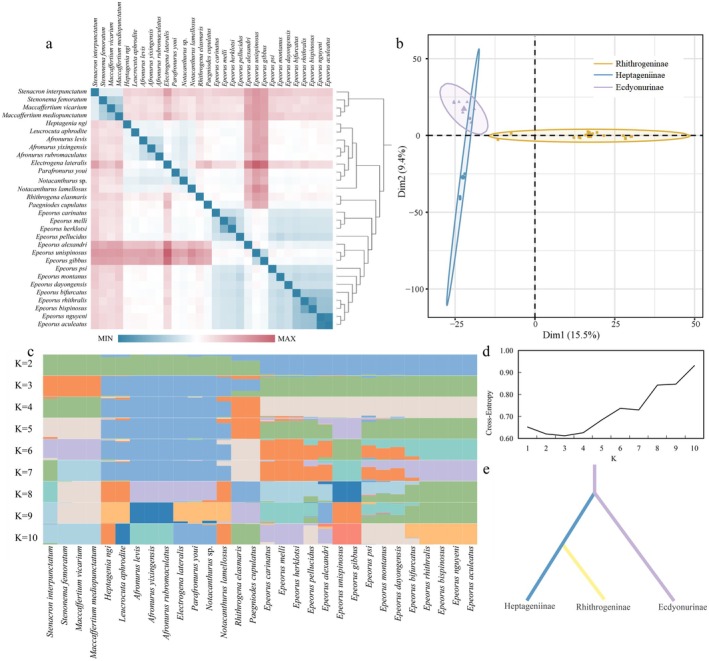
Analysis of Genetic Structure of Heptageniidae. (a) Genetic distance; (b) PCA analysis based on SNP; (c) Genetic component analysis; (d) Classification number and cross entropy; (e) Optimum species differentiation model.

Pairwise genetic distance analysis between *R. elasmaris* and other Heptageniidae species provided further insight into its phylogenomic placement and intergeneric differentiation (Figure 6a). The genetic distances between *R. elasmaris* and other genera within the family ranged from 0.226 to 0.272, indicating substantial molecular distinctness at the genus level. The smallest intergeneric distance (0.226) was observed between *R. elasmaris* and *Paegniodes cupulatus*, suggesting a relatively close phylogenomic affinity. Nevertheless, this value still exceeds the typical threshold of intrageneric divergence, supporting their classification as distinct genera. Within *Epeorus*, pairwise genetic distances varied considerably, ranging from 0.010 (*Epeorus nguyeni* vs. *Epeorus aculeatus*) to 0.238 (*Epeorus unispinosus* vs. *Epeorus alexandri*), indicating significant genetic heterogeneity or underlying taxonomic complexity within this genus.

The PCA further supported a structured genetic pattern among Heptageniidae species (Figure 6b). The first two principal components (PC1 = 15.5%, PC2 = 9.4%) collectively explained 24.9% of the total genetic variation. Species from the three subfamilies formed distinct and separate clusters, consistent with the current subfamilial classification framework in general.

Genetic structure analysis of species (Figure 6c) indicated that, based on cross‐entropy analysis, the optimal number of genetic clusters (*K*) was three (Figure 6d). At *K* = 3, the genetic structure was highly consistent with the current classification into three subfamilies: the vast majority of species exhibited a single, distinct subfamily‐specific genetic component, indicating significant genetic differentiation among the subfamilies. However, two notable exceptions were identified: *R. elasmaris* and *Paegniodes cupulatus*. In these two species, admixed genetic components from all three subfamilies were detected. Notably, the mitochondrial genomes of these two species showed a higher proportion of genetic components derived from the Ecdyonurinae subfamily compared to their own putative subfamily, Rhithrogeninae. Additionally, *Heptagenia ngi* possesses the genetic components of Ecdyonurinae, rather than Heptageniinae.

The species divergence model (Figure 6e) suggested that the most likely diversification pattern among the three subfamilies involved an initial divergence between Ecdyonurinae and Heptageniinae, followed by the divergence of Rhithrogeninae from the lineage of Heptageniinae.

### 3.4 Phylogenomic

**FIGURE 7:**
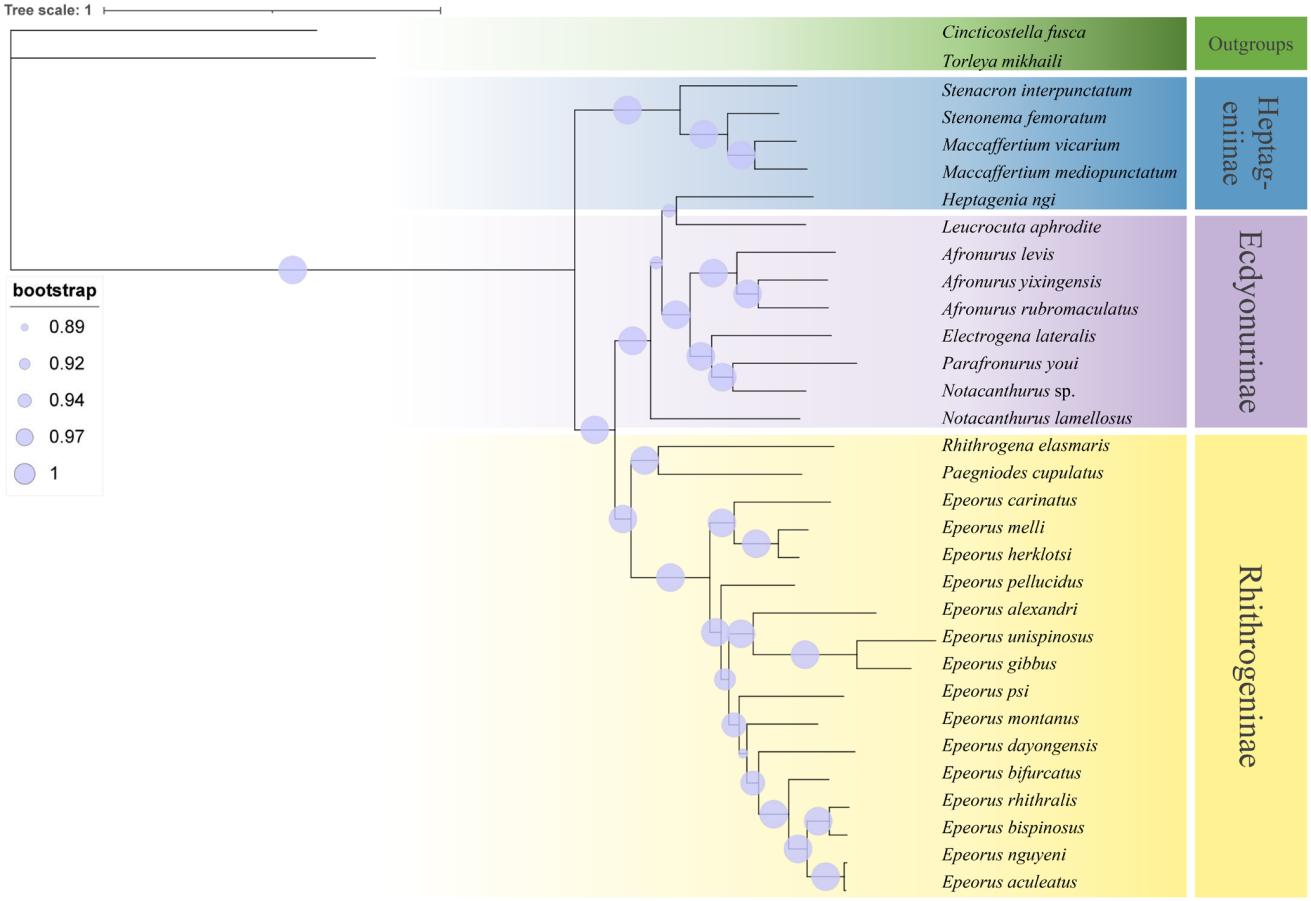
BI tree of Heptageniidae based on mitochondrial genome.

Phylogenomic relationships within Heptageniidae were reconstructed using Bayesian Inference (BI) based on the complete mitochondrial genomes (Figure 7). The topology revealed two major clades: one comprising Heptageniinae (excluding *Heptagenia ngi*), and the other consisting of Ecdyonurinae and Rhithrogeninae. *Heptagenia ngi* has been assigned to Ecdyonurinae instead of Heptageniinae. Within Rhithrogeninae, *R. elasmaris* was placed firmly within the lineage, forming a highly supported sister relationship with *Paegniodes cupulatus* (posterior probability, PP = 1.00). Together, these two species constituted a sister clade to *Epeorus*. The *Paegniodes‐R. elasmaris* clade occupied a basal position within Rhithrogeninae, supporting the hypothesis that *Paegniodes* represents an early diverging lineage in this subfamily. Within *Afronurus*, *A. levis* , *A. yixingensis*, and *A. rubromaculatus* formed a strongly supported monophyletic group (PP = 1.00). Overall, the inferred phylogeny corroborates the subfamilial phylogenomic framework proposed by Xu et al. (2021), summarized as (Heptageniinae + [Ecdyonurinae + Rhithrogeninae]).

## 4 Discussion

### 4.1 Natural Selection Dominates the Evolution and Structural Characteristics of the *Rhithrogena elasmaris* Mitochondrial Genome

The mitochondrial genome of *R. elasmaris* exhibits a pronounced compositional bias, characterized by negative AT‐skew (−0.264 to −0.052) and GC‐skew (−0.347 to −0.267). Such asymmetry typically reflects strand‐specific mutational pressures during replication and/or transcription. According to the traditional replication‐associated asymmetry (RAA) model, prolonged single‐stranded exposure of the heavy strand (H‐strand) during replication favors adenine deamination, resulting in T and G enrichment on the H‐strand (Touchon et al. 2005). However, in *R. elasmaris*, the dominant substitution patterns (T > C and A > G) contradict this prediction, suggesting that replication alone cannot fully explain the observed strand asymmetry.

The PR2‐, ENC‐, and neutrality‐plot collectively indicate that natural selection plays a predominant role in shaping codon usage patterns. Quantitative estimates from the neutrality plot reveal that mutational pressure accounts for only 24.14% of base compositional variance, whereas natural selection contributes 75.86%. Selective pressure likely acts on multiple molecular traits, including maintenance of RNA secondary structure stability, optimization of translation efficiency, or the facilitation of mitochondrial‐nuclear genome coadaptation (Wang et al. 2025; Zhou et al. 2025). These results suggest that the *R. elasmaris* mitochondrial genome is predominantly shaped by natural selection, representing a substantial shift in the mutation‐selection equilibrium toward the selection‐dominated end of the spectrum.

Within the compact 15,326 bp mitochondrial genome, 12 SSRs were identified, predominantly distributed across tRNA genes, intergenic regions, or at coding gene junctions. Different from the frequently observed enrichment of SSRs in the control region, SSRs in *R. elasmaris* are dispersed throughout the genome (Li et al. 2002). Trinucleotide repeats constitute the majority (7 of 12), implying that strand‐slippage replication could generate length polymorphism that modulates the expression of flanking genes (Ellegren 2004). Furthermore, although SSR insertions or deletions have the potential to cause frameshift mutations or premature termination, no such events creating independent ORFs were detected in this study. This implies that deleterious SSR variations have been removed by purifying selection, retaining only tolerable variants (Feng et al. 2022).

Codon usage bias analysis identified 27 high‐frequency codons (RSCU > 1), of which 25 terminate with A or U. Notably, the UUA (Leu) codon exhibited the highest RSCU value (2.54), aligning with the widespread AT‐ending codon preference widely observed in insect mitochondrial genomes (Herbeck and Novembre 2003). The pronounced bias toward leucine codons suggests that the corresponding tRNA‐Leu (UUR) gene may have undergone copy number amplification or anticodon modification, which may co‐evolve with this translational preference (Webster et al. 2017).

All 22 tRNAs retain the typical cloverleaf structure, yet display non‐canonical base pairings in all four structural arms. Although these mismatches may reduce thermodynamic stability, they likely enhance structural plasticity and recognition accuracy by aminoacyl‐tRNA synthetases (Niu et al. 2024; Naganuma et al. 2014). Thus, *R. elasmaris* appears to balance between translational efficiency and fidelity through nuanced structural adjustments rather than strict sequence conservation. This strategic balance between plasticity and stability may provide a selective advantage during the larval stage, which is characterized by rapid development and elevated metabolic demand.

Moreover, the codon usage correspondence analysis (COA) revealed that the first four principal axes explained only 65.97% of total variance, with genes distributed across the ordination space. This pattern suggests that codon usage bias is governed by the interplay of multiple factors. Beyond the aforementioned selective pressures, processes such as mito‐nuclear coevolution, RNA modifications, and thermal adaptation may also account for the remaining variation (Wang et al. 2025). In particular, mitochondrial tRNAs modifications such as 5‐taurinomethyluridine can alter codon–anticodon pairing rules and indirectly drive codon preference (Suzuki et al. 2020). Given that *R. elasmaris* inhabits cold‐water stream environments, where the stability of RNA secondary structures is critical at low temperatures, selection may also favor AU‐rich codons to reduce folding free energy (Rissone et al. 2024).

Under low‐temperature conditions, the stability of RNA secondary structures is crucial for translational efficiency. AU base pairs have lower melting free energy than GC pairs; selection may favor A/U‐ending codons to maintain tRNA–mRNA interaction stability (Rissone et al. 2024). The high metabolic demands and rapid development characteristic of mayfly larval stages may also drive codon bias through translational efficiency maximization mechanisms, adapting to energy‐intensive life phases (Webster et al. 2017). Several analyses of *R. elasmaris* imply a possible relationship in which changes in gene encoding respond to environmental pressures. Here, we attempt to assume that habitat may play a remarkable role in the ecology, metabolism, development, and physiological adaptations of *R. elasmaris*, which typically inhabit cold‐water lotic streams, fluctuating dissolved oxygen levels, and large lamellar gills, further reinforcing natural selection for optimized codon usage (He et al. 2022). Future research integrating physiological and phenotypic analyses (Zhou et al. 2026) could help to explain the evolutionary mechanism in codon usage bias of *R. elasmaris*.

### 4.2 The Evolutionary Pattern of the Mitochondrial Genome in Heptageniidae

The mitochondrial genomes of Heptageniidae exhibit a high degree of synteny at the macrostructural level, with no evidence of large‐scale gene rearrangements to date, consistent with the generally conserved evolutionary framework observed in metazoan mitochondria (Detcharoen et al. 2025). However, a non‐lineage‐specific loss of one of the multiple copies of *trnM* has been detected in several species. Such gene loss may arise from the activity of transposable elements, DNA repair mechanisms, and/or random genetic drift (Xia et al. 2017; Min et al. 2023). Given the inherent structural plasticity of tRNA tertiary architecture, which tolerates variations in arm length, this insertion neither disrupts the transcriptional polarity of the flanking genes nor incurs high adaptive costs. It is therefore plausible that this structural variant is evolving neutrally and has yet to be eliminated by purifying selection.

In terms of genetic diversity, the Heptageniidae mitogenomes exhibit a notably high average nucleotide diversity (Pi = 0.223), exceeding that of most insect groups (Zhang et al. 2021). This elevated diversity likely reflects repeated population bottlenecks and expansion events during glacial–interglacial cycles, or the long‐term maintenance of a large effective population size in this family (consistent with long‐term observation), which aligns with its observed extensive geographic distribution (Song 2016). As expected, PCGs are more conserved than non‐coding regions, consistent with the general evolutionary principle that non‐coding regions accumulate mutations more readily.

Among the PCGs, substantial heterogeneity in conservation levels was detected. nad6 displayed the highest nucleotide diversity (Pi = 0.357), whereas cox1 was the most conserved (Pi = 0.186), reflecting gene‐specific functional constraints. The exceptional conservation of *cox1*, *cox2*, and *cox3* stems from their critical role in the terminal step of the electron transport chain. The CuB‐heme a3 binuclear center of cytochrome c oxidase requires precise structural integrity; thus, mutations that impair proton pumping efficiency are efficiently eliminated by selection (Tsukihara et al. 1995). By contrast, Complex I (NADH dehydrogenase), characterized by structural flexibility and the presence of alternative electron input pathways, exhibits greater tolerance to mutations, thereby accumulating higher diversity (Čermáková et al. 2021). Correspondingly, the overall Pi value for the *nad* gene family (0.262) was significantly higher than that of the *cox* family (0.190), further supporting the positive correlation between functional importance and sequence conservation.

Selection pressure analysis revealed an average Ka/Ks ratio of 0.062 across the 13 PCGs, substantially < 1, indicating that these genes have been subjected to strong purifying selection throughout species divergence to preserve their core protein functions. However, the intensity of selection varied among individual genes. cox1 exhibited the lowest Ka/Ks value (0.0135), suggesting possible additional evolutionary constraints from nuclear‐encoded subunits (e.g., *cox4*, *cox5A*), which may form a nuclear–mitochondrial co‐evolutionary unit. Conversely, *atp8* showed the highest Ka/Ks ratio (0.187), consistent with its position within the Fo subcomplex and its role in modulating ATP synthesis rates. Although the *nad* gene family exhibited elevated sequence diversity, all members retained Ka/Ks below 1, indicating that critical functional sites remain subject to strong purifying selection (Wang et al. 2025).

The mitochondrial genomes of Heptageniidae have evolved under a macro‐syntenic framework via tRNA insertions and divergent selection pressures among PCGs. This framework provides mechanistic insight into the maintenance of energy homeostasis in aquatic insects facing extreme variations in temperature and dissolved oxygen, and establishes a theoretical basis for the application of mtDNA markers in biogeographic surveillance and phylogeny of mayflies. Elucidating the molecular drivers of mitochondrial adaptation will require future approaches that combine long‐read sequencing, correlated nuclear‐mitochondrial transcriptomic analyses, and CRISPR‐based functional assays.

### 4.3 Genetic Structure and Phylogenomic of Heptageniidae

Molecular evidence strongly supports the current subfamilial classification of Heptageniidae, yet simultaneously reveals conflicts between morphological delineations and mitochondrial evolutionary trajectories. The trans‐subfamilial genetic admixture observed in *R. elasmaris* and *Paegniodes cupulatus* underscores the limitations of relying solely on morphological traits for systematic delineation. Genetic component analysis indicates a dominant proportion of Ecdyonurinae‐derived elements in the mitochondrial genomes of these two species, suggesting that extensive mitogenomic sampling and sequencing of *Rhithrogena* and *Paegniodes* species may hold significant implications for understanding the origin and diversification of Heptageniidae. These patterns may imply potential incomplete lineage sorting or ancient mitochondrial introgression events, rather than recent hybridization (Li et al. 2023). However, given the maternal inheritance of mitochondrial genomes, further validation using nuclear genomic data is necessary. A preliminary genome survey based on low‐coverage NGS data (Figure S1) revealed that *R. elasmaris* is diploid, with an estimated genome size of approximately 791.16 Mb, relatively low repetitive element content, and a structurally simple genomic architecture, indicating relatively low difficulty for future genomic studies. phylogenomic and genetic structure analyses indicate that *Heptagenia ngi* is more closely related to Ecdyonurinae species, suggesting that its taxonomic status may need revision; it is possible that the specimen was misidentified. Within the genus *Epeorus*, interspecific mitochondrial divergence ranged from 0.010 to 0.238, exceeding conventional genus‐level thresholds. This exceptional divergence suggests potential paraphyly or the presence of cryptic lineages within *Epeorus*. Future studies integrating ecological niche modeling and morphological re‐examination will be essential to determine whether *Epeorus* should be subdivided into monophyletic units, ensuring that taxonomic designations accurately reflect evolutionary history. The reconstructed phylogeny supports the subfamilial framework proposed by Xu et al. (2021), i.e., (Heptageniinae + [Ecdyonurinae + Rhithrogeninae]). Within Rhithrogeninae, *Paegniodes cupulatus* and *R. elasmaris* form a strongly supported basal clade (PP = 1.00), diverging sequentially prior to *Epeorus* lineages. This topology supports the hypothesis that *Paegniodes* represents an early branching lineage within Rhithrogeninae (Zhang 2021). The basal position of *R. elasmaris*, coupled with its Ecdyonurinae dominated mitochondrial composition, further highlights the asynchronous evolution between mitochondrial lineages and morphological lineages. Furthermore, the subfamily Heptageniinae may not be monophyletic. Additional taxon sampling will be essential to evaluate the subfamily boundaries.

Based on the current phylogenomic framework, the most plausible diversification scenario for the three subfamilies of Heptageniidae involves an initial divergence between Ecdyonurinae and Heptageniinae, followed by the divergence of Rhithrogeninae from the Heptageniinae lineage. However, because this inference assumes subfamily monophyly, and the genetic composition of *Paegniodes cupulatus* and *R. elasmaris* remains complex, the exact diversification history warrants further investigation using integrative phylogenomics.

This study provides a robust phylogenomic framework for Heptageniidae at the subfamilial level, yet it simultaneously exposes the taxonomic ambiguities and unresolved relationships that persist within the family. A comprehensive revision will require expanded genomic sampling and morphological reevaluation, which together will refine our understanding of Heptageniidae diversification and evolutionary history.

## Author Contributions

**Qi‐Yong Mu:** conceptualization (equal), data curation (equal), formal analysis (equal), investigation (equal), methodology (equal), software (equal), writing – original draft (equal). **Quan Zhou:** data curation (equal), methodology (equal), validation (equal), visualization (equal), writing – original draft (equal). **Shook Ling Low:** methodology (equal), writing – review and editing (equal). **Yong‐Jing Zhao:** data curation (equal), investigation (equal). **Yong‐Xia Liu:** project administration (equal), resources (equal). **Jun‐Yan Wu:** data curation (equal), formal analysis (equal), writing – review and editing (equal). **Yong‐De Cui:** writing – review and editing (equal).

## Funding

This study was supported by the “Western Light” visiting scholar program (2024137) and the Guizhou Provincial Ecological Environmental Science and Technology Project of China (202335).

## Conflicts of Interest

The authors declare no conflicts of interest.

## Supporting information


**Figure S1:** Genome survey of *Rhithrogena elasmaris*.

## Data Availability

The assembled mitochondrial genome sequence from this study has been deposited in NCBI under the accession number PX826412.
