# Supplementary figures and images for: Characterization and Phylogenomic Implications of the Mitochondrial Genome of Rhithrogena elasmaris (Ephemeroptera: Heptageniidae)
Source: Ecol Evol. 2026 Feb 12;16(2):e73034. doi: 10.1002/ece3.73034 (PMC12901674; doi:10.1002/ece3.73034)

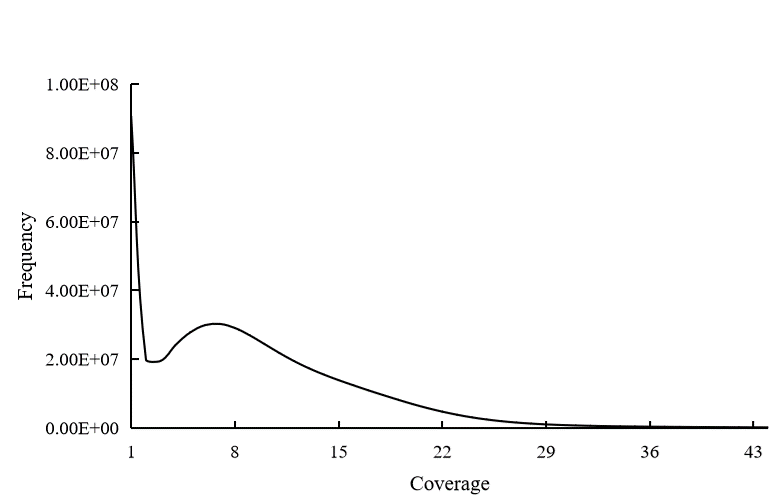

Supplement: Supplementary file 1 — Figure S1: Genome survey of Rhithrogena elasmaris. [file ECE3-16-e73034-s001.png]
